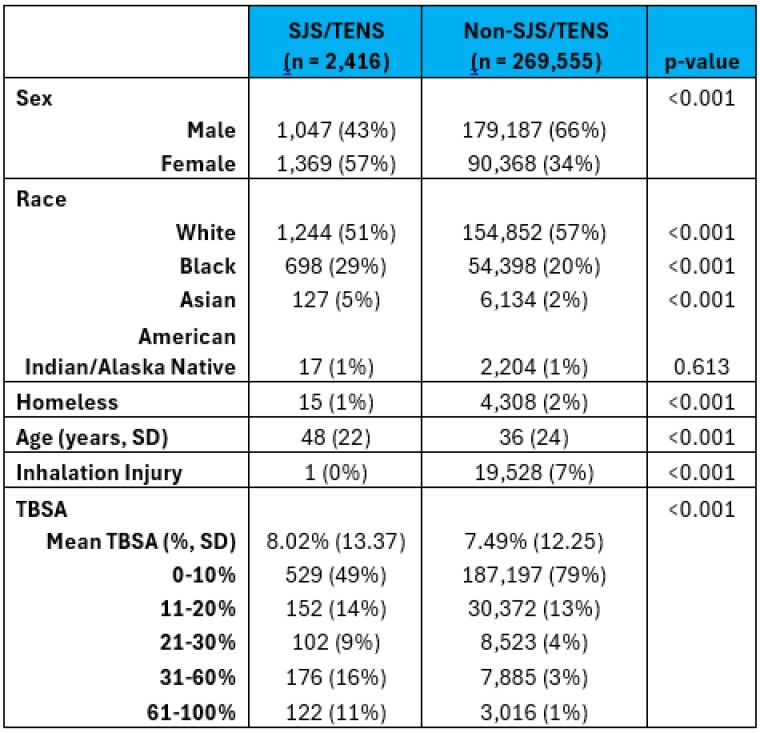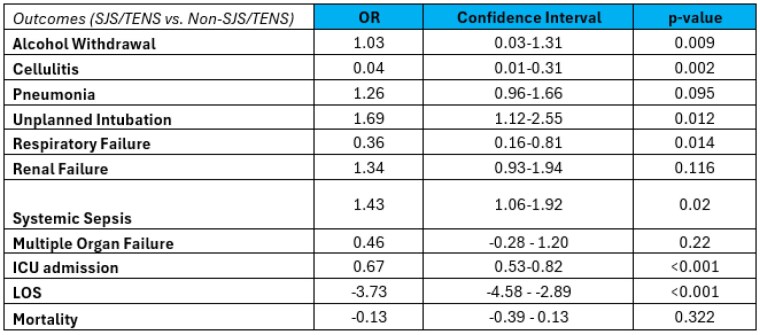# 581 The Outcomes of SJS/TENS: A Nationwide Analysis

**DOI:** 10.1093/jbcr/iraf019.210

**Published:** 2025-04-01

**Authors:** Tyler Murphy, Arman Fijany, Emily Swafford, Jordan Garcia, Punit Vyas, Robel Beyene, Stephen Gondek, Anne Wagner, Elizabeth Slater

**Affiliations:** Vanderbilt Burn Center; Vanderbilt University Medical Center; Vanderbilt Burn Center; Vanderbilt University Medical Center; Vanderbilt University Medical Center; Vanderbilt University Medical Center; Vanderbilt University Medical Center; Vanderbilt University Medical Center; Vanderbilt University Medical Center

## Abstract

**Introduction:**

Stevens-Johnson Syndrome (SJS) and Toxic Epidermal Necrolysis Syndrome (TENS) is a rare but potentially fatal skin reaction often accompanied by multi-system organ dysfunction. However, large-scale studies are limited due to its rarity. These patients are frequently cared for in burn units, with outcomes captured in American Burn Association (ABA) Noncommercial Burn Research Dataset (NBR). We hypothesize that patients with SJS/TENS will have higher rates of respiratory and infectious complications in comparison to their burn patient counterparts.

**Methods:**

This retrospective cohort study included adults from the 2012-2021 NBR looking at patients admitted with a diagnosis of SJS/TENS. Patient demographics and outcomes were compared with and without SJS/TENS and adjusted for age, sex, inhalation injury and percent total body surface area (TBSA). Logistical regression was used for the binary outcomes including alcohol withdrawal, cellulitis, pneumonia, unplanned intubation, respiratory failure, renal failure, systemic sepsis, multiple organ failure, ICU admission, and mortality. Linear regression was used for the continuous outcome of hospital length of stay (LOS).

**Results:**

Of 271,971 patients queried within the NBR, 2,416 patients had a diagnosis of SJS/TENS. These patients were statistically more likely to be older (mean 48 years [SD 22] vs. 36 years [24]), female (57% vs. 34%), not homeless (1% vs. 2%) and have a higher mean TBSA (8.02% [13.37] vs. 7.49% [12.25]). In multivariable analysis, SJS/TEN was associated with increased risk of cellulitis (odds ratio [OR] 0.04; 95% confidence interval [CI] 0.01,0.31), unplanned intubation (OR 1.69; CI 1.12, 2.55), respiratory failure (OR 0.36; CI 0.16-0.81), and sepsis (OR 1.43; CI 1.06, 1.92). Patients within the SJS/TENS cohort were more likely to experience alcohol withdrawal during their admission (OR 1.03; 0.03,1.31). Patients were significantly more likely to need an ICU admission (OR 0.7; CI 0.525,0.815) but had a shorter hospital length of stay (OR -3.7; CI -4.575, -2.885). There was no significant change in mortality.

**Conclusions:**

SJS/TENS is a rare but extremely morbid condition as supported by these results showing increased unplanned intubations and respiratory failure as well as sepsis. Some limitations to this study are the potential for misclassification or missing variables within a national data set. Further research is needed to investigate the association found by our data between alcohol use and SJS/TENS and practices to better protect patients from complications associated with SJS/TENS.

**Applicability of Research to Practice:**

This is one of the largest and first nationwide database projects to investigate the complications and outcomes of SJS/TENS and their comparative frequency relative to other burn populations treated by the practicing burn physician.

**Funding for the Study:**

N/A